# Rapid implicit extraction of abstract orthographic patterns of Chinese characters during reading

**DOI:** 10.1371/journal.pone.0229590

**Published:** 2020-02-21

**Authors:** Xiaochen Zhang, Siqin Yang, Minghu Jiang

**Affiliations:** 1 Shanghai Mental Health Center, Shanghai Jiao Tong University School of Medicine, Shanghai, China; 2 Center for Psychology and Cognitive Science, Tsinghua University, Beijing, China; 3 Lab of Computational Linguistics, School of Humanities, Tsinghua University, Beijing, China; University of Oslo, NORWAY

## Abstract

Orthographic processing is crucial in reading. For the Chinese language, sub-lexical processing has already taken place at radical level. Previous literature reported early position-specific radical representations and later position-general radical representations, implying a possible separating process of abstract position information irrespective of radicals *per se* from radical representations during orthographic processing. However, it remains largely unclear whether the abstract pattern of spatial arrangement of radicals can be rapidly extracted, and if so, whether this extraction takes place at the visual cortex, the very first processing center. As the visual cortex is documented to actively participate in orthographic processing, it may also play a role in the possible extraction of abstract orthographic patterns of Chinese characters. Hence, we hypothesize that abstract orthographic patterns of Chinese characters are covertly extracted at the visual cortex during reading. In this study, we investigated whether the visual cortex could rapidly extract abstract structural patterns of Chinese characters, using the event-related potential (ERP) technique. We adopted an active oddball paradigm with two types of deviant stimuli different only in one feature, structural or tonal, from standard stimuli; in each of the two sessions, subjects focused conscious attention on one feature and neglected the other. We observed that the ERPs recorded at occipital electrodes responded differentially to standard and structural deviant stimuli in both sessions, especially within the time range of the occipital P200 component. Then, we extracted three source waves arising from different levels of the visual cortex. Early response differences (from 88 to 456 ms after stimulus onset) were observed between the source waves, probably arising from left primary/secondary and bilateral associative visual cortices, in response to standard and deviant stimuli that violated abstract structural patterns, whether subjects focused their attention on the character structure or not. This suggests rapid extraction of abstract structural patterns of Chinese characters in the visual cortex, no matter the abstract structural pattern was explicit or implicit to subjects. Note that the source waves arising from right primary/secondary visual cortices in response to standard and structural deviant stimuli did not differ at all, indicating that this extraction of the abstract structural pattern of Chinese characters was left-lateralized. Besides, no difference was observed between source waves originating from any level of the visual cortex to standard and deviant stimuli that violated abstract tonal patterns, until 768 ms when a late effect related to conscious detection of targets occurred at higher levels of the visual cortex. Note that at late stages (later than 698 ms after stimulus onset), responses arising from bilateral associative visual cortices to standard and target stimuli differed for both sessions, no matter the structural or tonal feature was attended to. Our findings support the primitive intelligence of visual cortex to rapidly extract abstract orthographic patterns of Chinese characters that might be engaged in further lexical processing. Our findings also suggest that this rapid extraction can take place implicitly during reading.

## Introduction

As a critical carrier of communications among humans, the written language is considerably variable. It is marvelous for humans to be able to perceive written scripts and extract relevant features fast [1,2]. During reading, our literate brain is able to reach the speech form of words via dual routes, using mental lexicon or via grapheme-to-phoneme conversion [3]. Whichever route is taken, written scripts are first processed in the visual cortex in a hierarchical manner, with simple features like oriented bars detected first and more complex features like letter fragments and shapes detected later [4]. Eventually, abstract word representations may be formed at the visual word form area in the fusiform gyrus [5,6].

It is a key step to combine units of written scripts into a whole during the lexical processes before word representations are formed [4]. For example, letters are perceived and then bound into strings that form morphemes for alphabetic languages like English, and strokes into radicals for logographic languages like Chinese. In fact, there is evidence that sub-lexical processes have already occurred at the level of strings or radicals [7,8]. Their representations may assist the recognition of the whole word or character where the strings or radicals are embedded, supported by facilitatory priming effects when primes and targets share common strings or radicals [7]. Nevertheless, the further combination of strings and radicals differs in the way they are organized. For alphabetic languages, this combination usually takes place in a single dimension, combining strings into words. In contrast, for logographic languages, the combination of radicals takes place on a two-dimension plain. In the Chinese language, radicals can be bound in various manners. For example, they can be bound horizontally, forming a character with left-right structure; they can also be bound vertically, forming a character with up-down structure. Although there are other manners for radicals to be combined, over 86% of modern Chinese characters are with left-right (~65%) or up-down structure (~21%) [9,10].

Radical position information seems to be encoded and represented during orthographic processing. Although behavioral studies provide evidence supporting the emergence of both position-specific and position-general radical representations in Chinese character recognition [11,12], the time course of the covert processing underlying Chinese character recognition can only be revealed with neurophysiological techniques, e.g. the event-related potential (ERP) technique. For instance, Yum et al. found that pseudo-characters with illegally-positioned radicals elicited different occipital P100 and N170 from those with legally-positioned radicals, suggesting early encoding of radical position legality [13]. Similarly, Wei et al. discovered that the radical position legality in real characters and pseudo-characters with legally-positioned radicals was rapidly detected in native speakers as early as 120–160 ms reflected by ERP effects at frontal and central areas [14]; they also found that the brain of native speakers is able to differentiate real characters from pseudo-characters with legally-positioned radicals reflected by ERP effects at 170–210 ms [14], suggesting further lexical processing on the basis of radical position information. In addition, using a masked priming paradigm, Su et al. found that the target characters following the priming characters with different radical positions elicited larger occipital P100 (120–180 ms) and that the target characters with radicals appearing in their uncommon positions elicited larger N170 and P200 (225–325 ms), implying the early encoding of radical position specification and preference [15]. Furthermore, as to the differences between position-specific radical representations and more abstract position-general ones, Wu et al. provided ERP evidence supporting that the time courses for their emergences may differ, and that position-specific radical representations tended to be reflected by an ERP effect earlier than position-general ones [16].

The emergence order of position-specific and position-general radical representations inevitably suggests a possible abstracting process that isolates representations of radicals *per se* and representations of abstract radical positions. In alphabetic languages, the separation of letter identity and within-word letter position is crucial for subsequent semantic and phonological processing; the letter identity assists semantic extraction and the position information is necessary for representations of within-word-position-coded letter identities that will be utilized in phonological processing [17]. Since the semantic and phonological features are also processed at the radical level in Chinese character recognition [7,8], the separate representations for radicals *per se* and abstract radical positions may be more complex than in alphabetic languages. The role of the abstracted position information in orthographic processing of Chinese characters, to our surprise, was investigated by few studies. Representing the way that radicals of Chinese characters are combined, the abstract position information irrespective of radicals *per se* indeed affects the recognition of Chinese characters during reading. For instance, characters with left-right structure tend to be perceived faster than those with up-down structure [18]. In addition, since radicals are combined in various manners with different occurrence frequencies, it seems plausible that the literate brain of Chinese speakers may learn to extract rapidly the abstract spatial pattern of radical configuration embedded in a character during his/her long-term linguistic experience, so as to adapt to the linguistic surroundings and facilitate further processing to recognize Chinese characters. Therefore, the possible abstracting process that isolates representations of radicals *per se* and representations of abstract radical positions, the impact of the spatial arrangement of radicals on the recognition of characters, and the fact that the literate brain of Chinese speakers is continuously learning the statistics of the linguistic surroundings lead us to the hypothesis that the abstract pattern of spatial arrangement of radicals in Chinese characters are extracted during reading, probably in an implicit way.

To our knowledge, it remains largely unclear whether the abstract pattern of spatial arrangement of radicals irrespective of the radicals *per se* is extracted and represented during Chinese character recognition. As a part of orthographic processing, previous literature on the time course of orthographic processing for both alphabetic [19] and logographical languages [20,21] makes us hypothesize that the possible extraction of the abstract spatial pattern of radical configuration may take place at an early stage of Chinese character recognition, preceding phonological or semantic processing. We believe that this extraction process might take place after the emergence of position-specific radical representations [15,16], since the isolation of radicals *per se* and their positions, if existing, is likely to be involved as well in the extraction of the abstract spatial pattern of radical configuration. Previous literature also observed activations of a group of brain regions including fusiform gyrus and middle occipital gyrus (Brodmann areas 18, 19, and 37) specifically during orthographic processing [22,23], which may indicate the possible brain regions involved in the extraction of this abstract orthographic pattern of Chinese characters.

In this study, we attempted to explore whether the abstract pattern of spatial arrangement of radicals could be implicitly extracted during reading. We decided to use the ERP technique in order to track the time course of this covert procedure during orthographic possessing of Chinese characters. We were most interested in an ERP component named the visual mismatch negativity (vMMN), a response originating from the visual cortex and reflecting its predictive error of the statistics of the visual surroundings [24]. From an evolutionary perspective, our visual cortex evolves to detect sudden environmental change automatically, even if the change is subtle. When a series of standard stimuli is delivered to a subject, they would establish a norm of the statistics of the visual surroundings for his/her visual cortex to predict future stimuli. Then, when a rare, uncommon stimulus is presented out of sudden, his/her visual cortex would make a prediction error and thus generate the vMMN [24]. Therefore, the vMMN can be used to examine whether the visual cortex is able to perceive and recognize certain features of visual stimuli and, if so, can help to further detect the timing of the covert feature extraction. Previous literature on the vMMN suggests that the human visual system is not only sensitive to changes of such simple features of visual stimuli as color and line orientation [25,26], but also to violation of sequential regularity [27,28] and change of abstract features like gender category [29].

Importantly, the vMMN can also be elicited by violations of the regularity of lexical information. For instance, Wei et al. reported significant vMMN effects for surprising alternations between non-characters and real characters/pseudo-characters with legally-positioned radicals, among native Chinese speakers but not non-native speakers, suggesting that the brain of native Chinese speakers is capable of extracting lexical features with an early-triggered automatic process [14]. Their further study reported vMMN effects for deviant character frequency, suggesting that the statistics embedded in Chinese characters indeed affects early, automatic processing of Chinese characters [30]. Moreover, Wang et al. used the vMMN to study the rapid feature extracting procedure of Chinese characters during reading; they discovered significant vMMN effects for infrequent violations of the tonal pattern embedded in standard characters, indicating that abstract phonological patterns of Chinese characters are rapidly extracted in the visual cortex [31]. This vMMN effect still exists even when the stimuli are displayed at the corner of the screen away from the center to which the attention of subjects is attracted by an irrelevant visual task [32]. However, it still lacks evidence to clarify whether the visual cortex can implicitly extract abstract spatial patterns of radical configuration of Chinese characters. The ability of the visual cortex to automatically extract abstract phonological patterns supports the extraction of higher-level abstract lexical features in the visual cortex. Since abstract structural patterns stem somewhat more directly from visual cues than tonal patterns and orthographic processing generally precedes phonological processing [20,21], it is conceivable that abstract structure patterns may also be extracted in the visual cortex. Therefore, in this study, we expected to observe significant vMMN effects for infrequent violations of the structural pattern embedded in standard characters, whether or not these violations were the targets for subjects to detect with conscious attention.

Here, we adopted an active dual-deviant oddball paradigm, simulating a scene of reading. To be more specific, the visual stimulus stream used in this experiment was composed of three types of Chinese characters: those with left-right structure and falling tone, those with left-right structure and rising tone, and those with up-down structure and falling tone (probability: 8:1:1). In one of the two sessions in the experiment, subjects were instructed to respond to the target characters with a deviant structure (up-down) from the standard one (left-right). By doing so, subjects explicitly identified the violations of the standard abstract pattern of spatial arrangement of radicals in this session. We expected to observe differences within in the time range of the vMMN between ERPs in response to standard stimuli and target ones, which represented the active extraction of the abstract structural pattern. In the other session with exactly the same stimulus presentation, subjects were instructed to respond to the target characters with a deviant tone (rising) from the standard one (falling). Then, we examined whether there were also differences within in the time range of the vMMN between ERPs in response to standard stimuli and non-target ones with a different structure that were targets in the structure identification session. We expected to observe similar difference effects on ERPs as in the structure identification session; if so, this meant that the differences in ERPs in response to changes in the abstract structure pattern of Chinese characters could be extracted implicitly during reading.

This paradigm was also used to replicate the findings that abstract phonological patterns of Chinese characters are implicitly extracted in the visual cortex [31,32]. In the session where subjects were instructed to identify the target characters with a deviant tone, we expected to observe differences within in the time range of the vMMN between ERPs in response to standard stimuli and target ones, which represented the active extraction of the abstract tonal pattern. In the other session where subjects were instructed to identify the target characters with a deviant structure, we expected to observe similar difference effects on ERPs as in the tone identification session.

## Materials and methods

### Subjects

Sixteen subjects (8 females; mean age ± SD: 23 ± 3 years) participated in the experiment. All subjects were native Chinese speakers with normal or corrected-to-normal vision. None of the subjects had history of neurological or psychiatric disorders. All subjects were paid for their time and gave written informed consent to the experimental protocol approved by the local Ethics Committee of Tsinghua University.

### Experimental protocol

**Fig 1:**
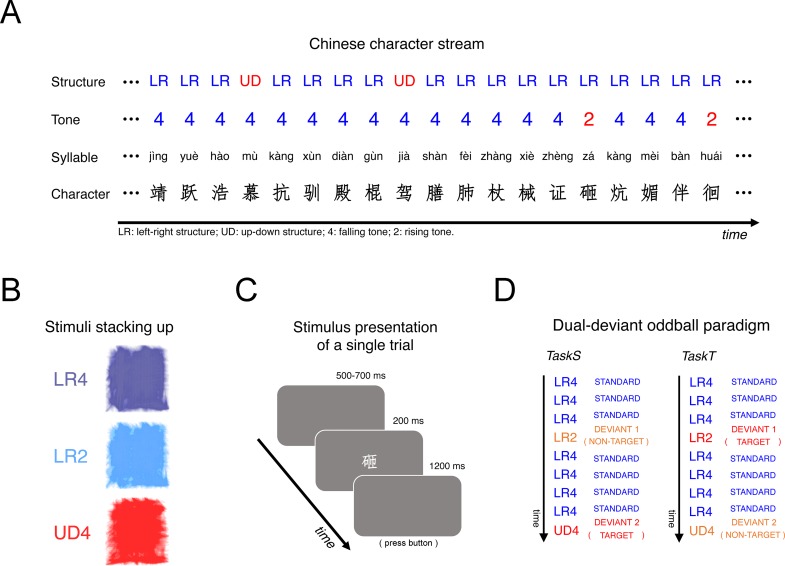
Experimental protocol and stimuli. (A) We adopted an active oddball paradigm with two types of deviant stimuli that violated abstract patterns of structural and tonal features of Chinese characters respectively. The standard stimuli were Chinese characters with left-right structure and falling tone (LR4). The two types of deviant stimuli were characters with up-down structure and falling tone (UD4) and characters with left-right structure and rising tone (LR2). The patterns of structural and tonal features abstract were abstract, since the characters were constructed with various radicals and their pronunciations contained various consonants and vowels. (B) Chinese characters stacking-up for the three stimulus types. Note they are considerably similar, suggesting minimal physical heterogenicity of visual stimuli. (C) The duration of the presence of each character was 200 ms and the stimulus onset asynchrony was 1.9–2.1 s. (D) The experiment included two sessions where exactly the same stream of characters was presented. In one session, subjects were instructed to respond to the deviant stimuli that violated the abstract structural pattern by pressing a button and neglected the other type (TaskS); in the other session, they were instructed to respond to the deviant stimuli that violated the abstract tonal pattern by pressing a button (TaskT).

We used an active oddball paradigm with two types of deviant stimuli, simulating a scene of reading (Fig 1A). In a stream of Chinese characters, characters with left-right structure and falling tone (LR4) occurred frequently as standard stimuli and two types of deviant stimuli were infrequently delivered between standard stimuli; one was characters with up-down structure and falling tone (UD4) and the other was characters with left-right structure and rising tone (LR2). Note that UD4 characters differed from LR4 characters only in structure and that LR2 characters differed from LR4 characters only in lexical tone. The characters were assembled with various radicals and their pronunciations contained various consonants and vowels, thus rendering the pattern of both structural and tonal features abstract.

The stream contained 1200 characters: 960 standard stimuli (LR4), 120 structural deviant stimuli (UD4), and 120 tonal deviant stimuli (LR2). Owing to the limitation of the number of real characters with left-right structure and falling tone, the stimulus pool contained 524 standard characters and 436 of them were presented twice. The characters were presented in a pseudo-random order, any two neighboring deviant stimuli were separated by at least two standard stimuli, and the standard characters that were delivered twice were not presented consecutively. All the stimuli were in white color, displayed at the center of a gray background. Subjects’ eyes were at the same height as the center of the screen and the eye-to-screen distance was ~1 m. The width and height of the Chinese characters were about 10 cm; they were all in Imitation Song typeface. The occurrence frequency and the stroke number of the characters were matched across stimulus types (Table 1); the occurrence frequency of the characters was obtained from the balanced corpus of modern Chinese of the State Language Commission of China (http://corpus.zhonghuayuwen.org). The final images of stacking-up stimulus characters for the three stimulus types were considerably similar, suggesting minimal physical heterogenicity of visual stimuli (Fig 1B). The duration of the presence of each character was 200 ms and the stimulus onset asynchrony was 1.9–2.1 s (Fig 1C). The delivery of stimuli was controlled by custom programs in E-Prime (2.0; Psychology Software Tools).

**Table 1:** The occurrence frequency and the stroke number of the stimuli.

		Number	Occurrence frequency (‰)		Stroke number	
			mean	SD	mean	SD
LR4		524	0.194	0.468	10	3
UD4		120	0.191	0.309	10	3
LR2		120	0.178	0.471	10	3
Variance homogeneity test			*p* = 0.613		*p* = 0.789	
ANOVA	F_2, 761_		0.067		0.207	
	*p* value		0.935		0.813	

The experiment included two sessions where exactly the same stream of characters was presented. In one session, subjects were instructed to respond to one type of deviant stimuli by pressing a button and neglect the other type; in the other session, they were instructed to respond to the other type of deviant stimuli. The session order was counterbalanced across subjects. As shown in Fig 1D, if the subjects focused their attention on the character structure, their task would be to detect the up-down structure (UD4) among a stream of characters with the left-right structure (LR4 and LR2); hereafter this session was referred to as TaskS. In TaskS, the target deviant stimuli were UD4 characters and the non-target deviant stimuli were LR2 characters. LR4 and LR2 characters together established an explicit abstract structural pattern (i.e. the left-right structure), and LR4 and UD4 characters together established an implicit abstract tonal pattern (i.e. the falling tone) to which subjects did not consciously attend. Similarly, if subjects focused their attention on the character tone, their task would be to detect the rising tone (LR2) among a stream of characters with the falling tone (LR4 and UD4); hereafter this session was referred to as TaskT. In TaskT, the target deviant stimuli were LR2 characters and the non-target deviant stimuli were UD4 characters. Here, the abstract tonal pattern (i.e. the falling tone) established by LR4 and UD4 characters was explicit, and the abstract structural pattern (i.e. the left-right structure) established by LR4 and LR2 characters was implicit. Intervals between stimuli were deliberately set short and subjects were instructed to respond as fast as possible, maximizing the psychological resources of the subjects devoted to the current task. In addition, the tone identification task was quite difficult. These ensured the detection of the unattended feature, especially the abstract structural pattern, to be implicit.

### Electrophysiological recording

During data acquisition, subjects were comfortably seated in an armchair. They were instructed to press a button in response to each target stimulus as quickly as possible. While they were doing the tasks, continuous electroencephalography (EEG) data were being recorded at a sampling rate of 500 Hz with a BrainAmp DC amplifier (Brain Products GmbH) and the software Recorder (1.20, Brain Products GmbH), using Ag/AgCl electrodes, simultaneously from 61 channels (Fp1/2, AF3/4/7/8, F1/2/3/4/5/6/7/8, FT7/8, FC1/2/3/4/5/6, T7/8, C1/2/3/4/5/6, TP7/8/9/10, CP1/2/3/4/5/6, P1/2/3/4/5/6/7/8, PO3/4/7/8, O1/2, Fpz, Fz, Cz, CPz, Pz, POz, Oz) according to the international 10/10 system, with the reference electrode placed at FCz and the ground electrode placed at AFz. Vertical and horizontal electro-oculograms were also recorded for elimination of ocular artifacts. Electrode impedance was maintained below 10 kΩ.

### Analysis of behavioral performances

A hit was recorded when the subject pressed the response button between 200 to 1400 ms after the onset of a target stimulus. The hit rate is the ratio of the number of hits to the number of target stimuli. The reaction time is the median time across trials between stimulus onset and the first hit. The indices were calculated in Matlab (R2013b, MathWorks) and compared between sessions with paired t-tests in SPSS (22.0.0, IBM); a priori significance level was set at 0.05.

### Pre-processing and analysis of event-related potentials

The pre-processing of event-related potentials (ERPs) included re-referencing, filtering, artifact elimination, bad-block rejection, segmenting, and averaging. First, the continuous EEG data were re-referenced to the average of all channels, and band-pass filtered between 1 and 70 Hz. Next, ocular and myogenic artifacts were largely eliminated with a data cleaning toolbox implanted in EEGLAB [33] on the basis of an EEG-cleaning algorithm named Artifact Subspace Reconstruction [34]; it automatically detects artifacts based on the principal component analysis and eliminated the artifacts based on clean parts of the data that had minimal noise. Afterwards, the continuous EEG data were segmented into epochs with duration of 1.25 s, including 250 ms pre-stimulus baseline. Then, all the epochs with data points exceeding ±80 μV were rejected, and all the valid epochs were averaged. The ERPs were baseline corrected, relative to the average of all pre-stimulus data (–250–0 ms).

We compared the ERPs in response to the standard and deviant stimuli with paired t-tests. The comparison was first conducted in sensor space; the ERPs recorded at occipital regions (PO3/4/7/8, O1/2) were averaged and further analyzed. A sliding window with duration of 40 ms moved with 2-ms step and the mean amplitude was compared between responses to the standard and the unattended deviant stimuli for each time point with paired t-tests. The *p* values were false discovery rate (FDR) corrected in order to control the overall Type I error rate. We also compared the ERPs in response to the standard and the target deviant stimuli, using the same analyzing approaches.

In order to compare the ERPs in response to different types of stimuli in source space, we extracted six components from the averaged ERPs using spatial filters that were obtained via the independent component analysis (ICA) [35]. The number of components was determined according to the explained variance of the retained components; we retained the fewest components that could explain at least 95% of the data variance. Spatial filters transformed the responses acquired on the scalp into responses arising from a functionally connected group of brain structures. Therefore, we assumed that each component represented the brain response arising from a configuration of functionally connected neural sources. We derived the source waves from original data for each subject, for each stimulus type, for each session.

In order to localize the neural sources for each spatial filter, we applied dipole fitting to the components derived from the ICA-based decomposition with DIPFIT 3.3 toolbox implanted in EEGLAB. We used a boundary element model derived from the MNI (Montreal Neurological Institute) averaged brain as the head model. The location, orientation, and strength of a couple of mirrored rotating dipoles (or a single dipole, for two components with lateralized topographies) were obtained through a scanning procedure aimed at minimizing the deviation between the estimated topography and the measured one [36]. The locations of the estimated equivalent dipoles were reported in the Talairach coordinate system. Residue variance (RV) was also reported to evaluate the accuracy of the source localization. We also reconstructed the sources underlying each component with exact low-resolution brain electromagnetic tomography (eLORETA). Different from dipole fitting, eLORETA uses a distributed source model for source reconstruction and provides exact, zero-error localization in the presence of measurement and structured biological noise [37]. The main aim for us to provide the results of source reconstruction based on eLORETA was to render the source reconstruction results from multiple methods comparable. Since the spatial resolution of the EEG data was generally low, the consistency between equivalent sources estimated through dipole fitting and eLORETA would render the source reconstruction results more convincing.

After the source waves were derived, we compared the ERPs in response to the standard and deviant stimuli in source space with paired t-tests. The comparing approach was the same as that for ERPs in sensor space. The ERP pre-processing, the ICA-based decomposition of ERPs, and the dipole fitting were conducted with the EEGLAB toolbox in Matlab, the eLORETA was conducted in the LORETA-KEY software package [38], and sensor- and source-level ERP analyses were conducted with custom programs in Matlab.

## Results

### Behavioral performances

**Fig 2:**
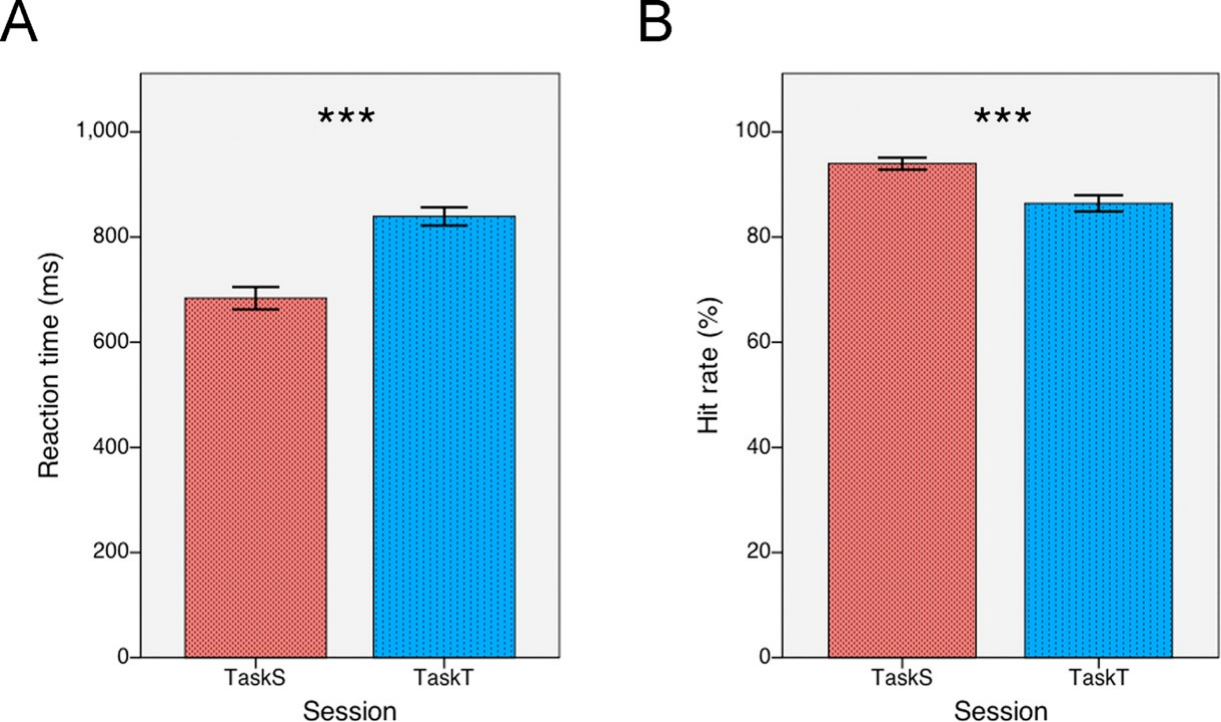
Behavioral performance. (A) The reaction time was significantly earlier for TaskS than for TaskT. (B) The hit rate was significantly higher for TaskS than for TaskT. Behavioral performance suggests that subjects detected structural features of Chinese characters faster and more accurately than tonal features when they focused their attention on the corresponding features. Error bars refer to ±SEM.

The mean reaction time was 684 ms for TaskS and 839 ms for TaskT (Fig 2A). A paired t-test showed that the reaction time was significantly shorter for TaskS than for TaskT (df = 15, t = 10.759, *p* < 0.001). The mean hit rate was 94% for TaskS and 86% for TaskT (Fig 2B). A paired t-test showed that the hit rate was significantly higher for TaskS than for TaskT (df = 15, t = 5.326, *p* < 0.001). This suggests that the tone identification task was more difficult than the structure identification task, ensuring the extraction of abstract structural pattern in TaskT to be implicit.

### Extraction of abstract structural pattern: Sensor-level analyses

**Fig 3:**
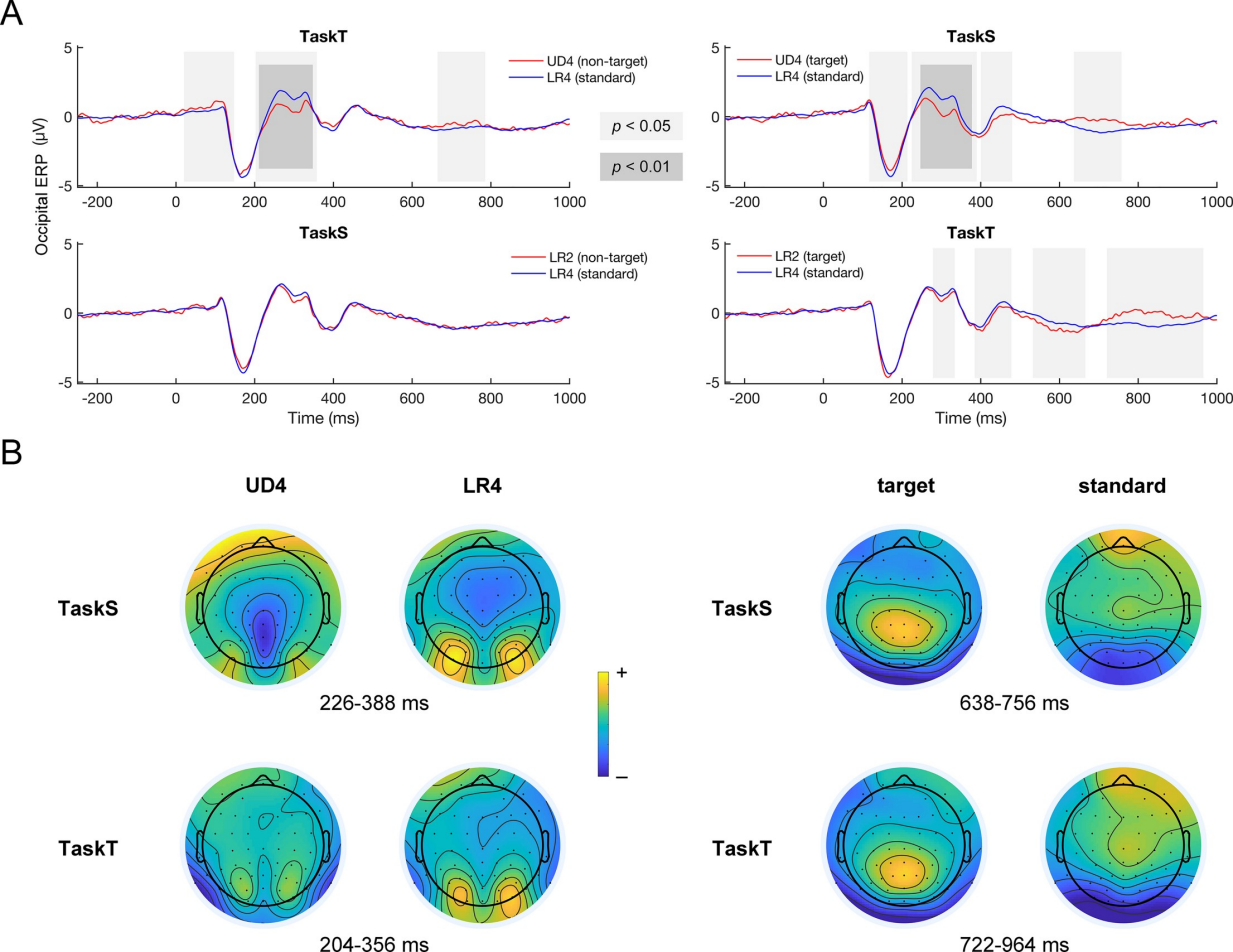
Occipital ERPs. (A) Responses recorded at occipital electrodes. Significant differences in occipital ERPs in response to standard and non-target stimuli in TaskT were observed, especially within the time range of P200. Similar response differences were also observed in TaskS between occipital ERPs in response to standard and target stimuli. These findings suggest that the abstract structural pattern could be detected explicitly and implicitly. In contrast, no significant difference in occipital ERPs in response to standard and non-target stimuli in TaskS was observed, but there were significant differences in occipital ERPs in response to standard and target stimuli in TaskT, suggesting that the abstract tonal pattern could only be detected if conscious attention was paid to the tonal feature of characters. (B) Topographies of the occipital P200 and parietal P300 components. The topographies of the occipital P200 component suggest that the difference effects indeed arose from the occipital lobe. The parietal P300 effects occurred primarily around Pz, although occipital ERPs also showed similar effects that may partially be a product of volume conduction.

We observed significant differences between occipital ERPs in response to standard (LR4) and target deviant stimuli (UD4) in TaskS. As shown in Fig 3A, the time range when the differences occurred was 118–212 ms (target deviant > standard), 226–388 ms (target deviant < standard), 402–478 ms (target deviant < standard), and 638–756 ms (target deviant > standard). This suggests that the abstract structural pattern of Chinese characters could be extracted rapidly when sufficient attentional resources were devoted to the character structure. Also observed were significant differences between occipital ERPs in response to standard (LR4) and non-target deviant stimuli (UD4) in TaskT. As shown in Fig 3A, the time range when the differences occurred was 22–146 ms (non-target deviant > standard), 204–356 ms (non-target deviant < standard), and 666–784 ms (non-target deviant > standard). Therefore, the differences between ERPs, especially the P200 component, in response to standard stimuli and structural deviant stimuli were significant, whether or not conscious attention was paid to the abstract structural pattern, suggesting that violations of the abstract structural pattern could be detected even in an implicit manner with conscious attention paid to the tonal features instead of structural ones. The topographies of the P200 component are given in Fig 3B, suggesting that the difference effects indeed arose from the occipital lobe; on the other hand, the late ERP differences largely represented P300 effects and may be related to target detection. The statistics of the mean amplitude of occipital ERPs within the time periods of interest were given in Table 2.

**Table 2:** Effects of occipital ERPs (scalp responses at PO3/4/7/8 and O1/2 collapsed) reflecting extraction of abstract structural or tonal pattern.

Session	Time period (ms)	Character	Mean amplitude (μV)		paired-t
			mean	SD	(df = 15)
TaskS	118–212	LR4	–2.4717	1.6270	3.5735 **
		UD4	–2.1096	1.4920	
	226–388	LR4	0.8576	1.1370	5.6384 ****
		UD4	0.1546	0.9487	
	402–478	LR4	0.1658	0.8945	2.9037 *
		UD4	–0.3514	0.9262	
	638–756	LR4	–1.0112	0.8155	3.9088 **
		UD4	–0.2125	0.6551	
TaskT	22–146	LR4	0.1131	0.7892	4.6392 ***
		UD4	0.4110	0.8078	
	204–356	LR4	0.9196	1.3813	6.1158 ****
		UD4	0.2881	1.4245	
	666–784	LR4	–0.8181	0.7447	4.2483 ***
		UD4	–0.5319	0.7024	
TaskT	280–332	LR4	1.4904	1.8384	2.6990 *
		LR2	1.1991	2.0012	
	386–476	LR4	–0.0006	0.7079	3.0223 **
		LR2	–0.2888	0.8871	
	534–664	LR4	–0.6538	0.6523	3.4566 **
		LR2	–1.1081	0.7002	
	722–964	LR4	–0.8039	0.9654	4.4300 ***
		LR2	–0.0805	0.8490	

**** *p* < 0.0001

*** *p* < 0.001

** *p* < 0.01

* *p* <0.05.

### Extraction of abstract tonal pattern: Sensor-level analyses

We observed significant differences between occipital ERPs in response to standard (LR4) and target deviant stimuli (LR2) in TaskT. As shown in Fig 3A, the time range when the differences occurred was 280–332 ms (target deviant < standard), 386–476 ms (target deviant < standard), 534–664 ms (target deviant < standard), and 722–964 ms (target deviant > standard). This suggests that the abstract tonal pattern of Chinese characters could still be extracted when sufficient attentional resources were devoted to the character tone, but this extraction was much slower than the extraction of the abstract structural pattern. According to the topography shown in Fig 3B, the late ERP differences probably represented the P300 component and were related to target detection. We did not observe any significant difference between occipital ERPs in response to standard (LR4) and non-target deviant stimuli (LR2) in TaskS, indicating that the extraction of the abstract tonal pattern required conscious attention paid to the character tone and probably could not take place implicitly. The statistics of the mean amplitude of occipital ERPs within the time periods of interest were given in Table 2.

### ERP decomposition and source localization

**Fig 4:**
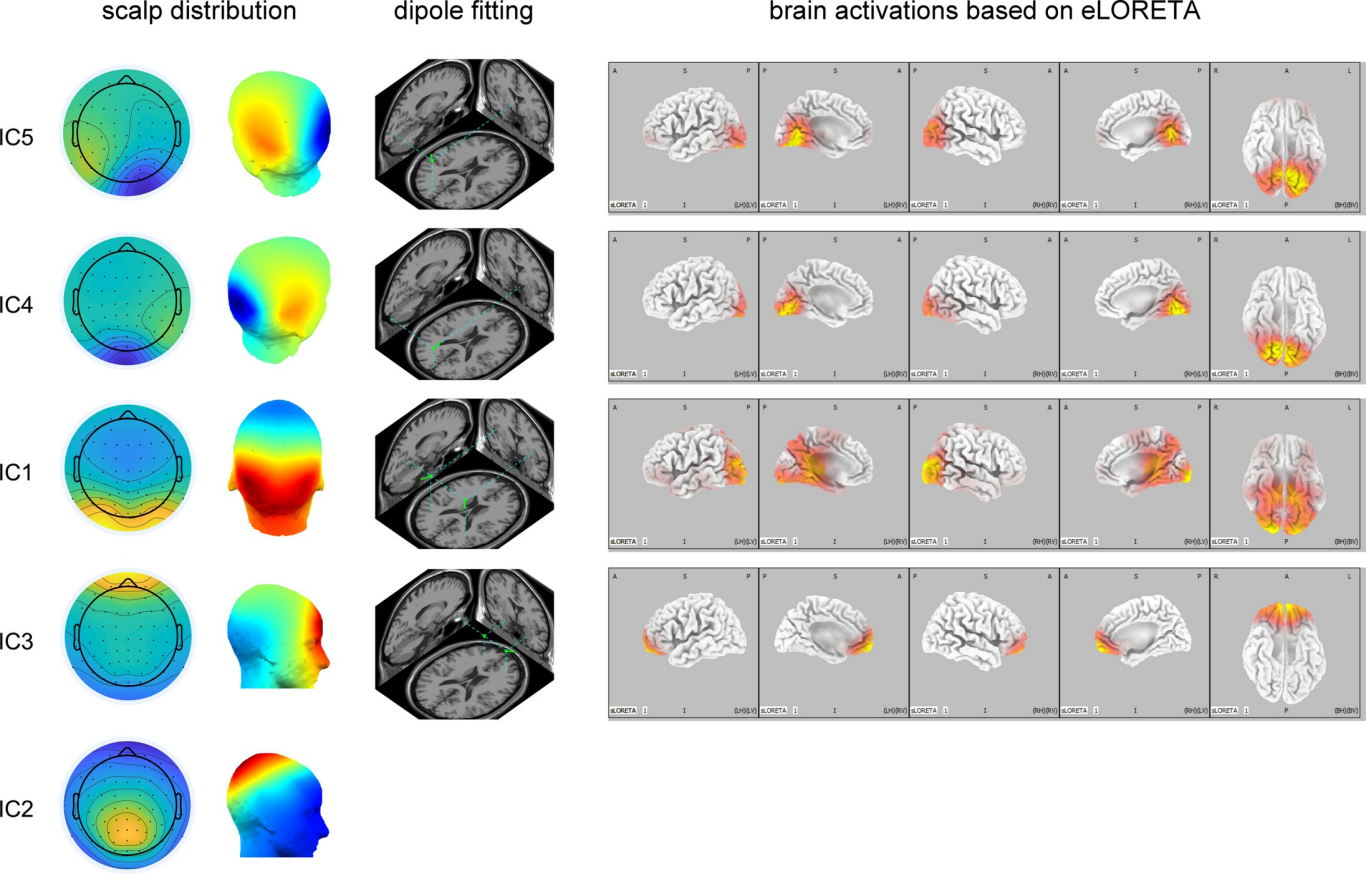
Source reconstruction results. The 2-D and 3-D topographies, the equivalent dipole(s) in an average brain, and the eLORETA results of each component are displayed. IC5 probably arose from the left primary/secondary visual cortices. IC4 probably arose from the right primary/secondary visual cortices. IC1 arose from a higher level of the visual cortex compared with IC5 and IC4, probably the bilateral associate visual cortices. IC3 probably arose from the orbitofrontal cortex (Brodmann area 11). IC2 clearly represented the P300 component.

We extracted six components that explained 95.18% of the whole data. Based on visual inspection, their topographies suggest that three components arose from the occipital lobe (IC5, IC4, and IC1; Fig 4), one from the frontal lobe (IC3; Fig 4), and one from the generating sites of P300 (IC2; Fig 4).

Further source localization suggested that the neural sources underlying IC5, IC4, and IC1 may be largely attributed to the left primary/secondary visual cortices, to the right primary/secondary visual cortices, and to the bilateral associate visual cortices and possibly regions responsible for higher-level linguistic processing, respectively. Supportively, the dipole fitting pinpointed the equivalent dipole underlying IC5 to [–6, –83, 4] mm (RV = 3.52%; Fig 4); the gray matter with which the cube centered at this coordinate (range: ± 4 mm) most largely overlapped was cuneus and lingual gyrus (Brodmann areas 17 and 18), lateralized to the left hemisphere. In addition, the dipole fitting pinpointed the equivalent dipole underlying IC4 to [7, –95, –14] mm (RV = 2.52%; Fig 4); the gray matter with which the cube centered at this coordinate (range: ± 4 mm) most largely overlapped was lingual gyrus (Brodmann areas 17 and 18), lateralized to the right hemisphere. Also, the dipole fitting pinpointed the pair of equivalent mirrored dipoles underlying IC1 to [±37, –55, 12] mm (RV = 0.92%; Fig 4); the gray matter with which the cube centered at these coordinates (range: ± 4 mm) most largely overlapped was middle and superior temporal gyrus (Brodmann areas 19 and 22) in both hemispheres.

The source reconstruction results based on eLORETA for these three components were consistent with the dipole fitting results. The equivalent sources for the three components were all largely distributed in occipital lobes, but at different parts. The sources underlying IC5 and IC4 were distributed in a more restricted range of areas and closer to the occipital poles compared with those underlying IC1 (Fig 4), largely overlapped with Brodmann areas 17 and 18. This suggests that IC4 and IC5 may represent neural activities at primary and/or secondary visual cortices. On the other hand, the sources underlying IC1 were distributed in a wider range and farther from the occipital poles (Fig 4), largely overlapped with Brodmann areas 18 and 19; some activated sources even reached Brodmann areas 37 and 39, the former of which was relevant to orthographic processing [22,23] and the latter of which was a part of Wernicke's area which plays vital roles in understanding written or spoken language. The distributions of brain activations underlying IC5, IC4, and IC1 revealed by eLORETA were given in Table 3. This suggests that IC1 may represent neural activities at a higher level of the visual cortex compared with IC5 and IC4.

**Table 3:** Percentage of brain activations at each Brodmann area (sum of all voxels whose activation was greater than 75% of the maximum of whole brain activation) for IC5, IC4, and IC1.

	Brodmann Area				
	17	18	19	37	39
IC5	26.5%	57.5%	15.9%	0	0
IC4	22.3%	67.5%	10.2%	0	0
IC1	6.4%	54.9%	27.0%	7.5%	4.2%

IC3 may arise from orbitofrontal cortex. The dipole fitting pinpointed the pair of equivalent mirror dipoles underlying IC3 to [±23, 42, –27] mm (RV = 14.10%; Fig 4); the gray matter with which the cube centered at this coordinate (range: ± 4 mm) most largely overlapped was Brodmann area 11 in both hemispheres. Source reconstruction based on eLORETA gave similar results (Fig 4). It was quite obvious that IC2 represented P300 (Fig 4); it had similar topography and response behavior to those of P300 reported in previous literature [39,40]. Hence, we did not localize the sources for IC2.

### Extraction of abstract structural pattern: Source-level analyses

**Fig 5:**
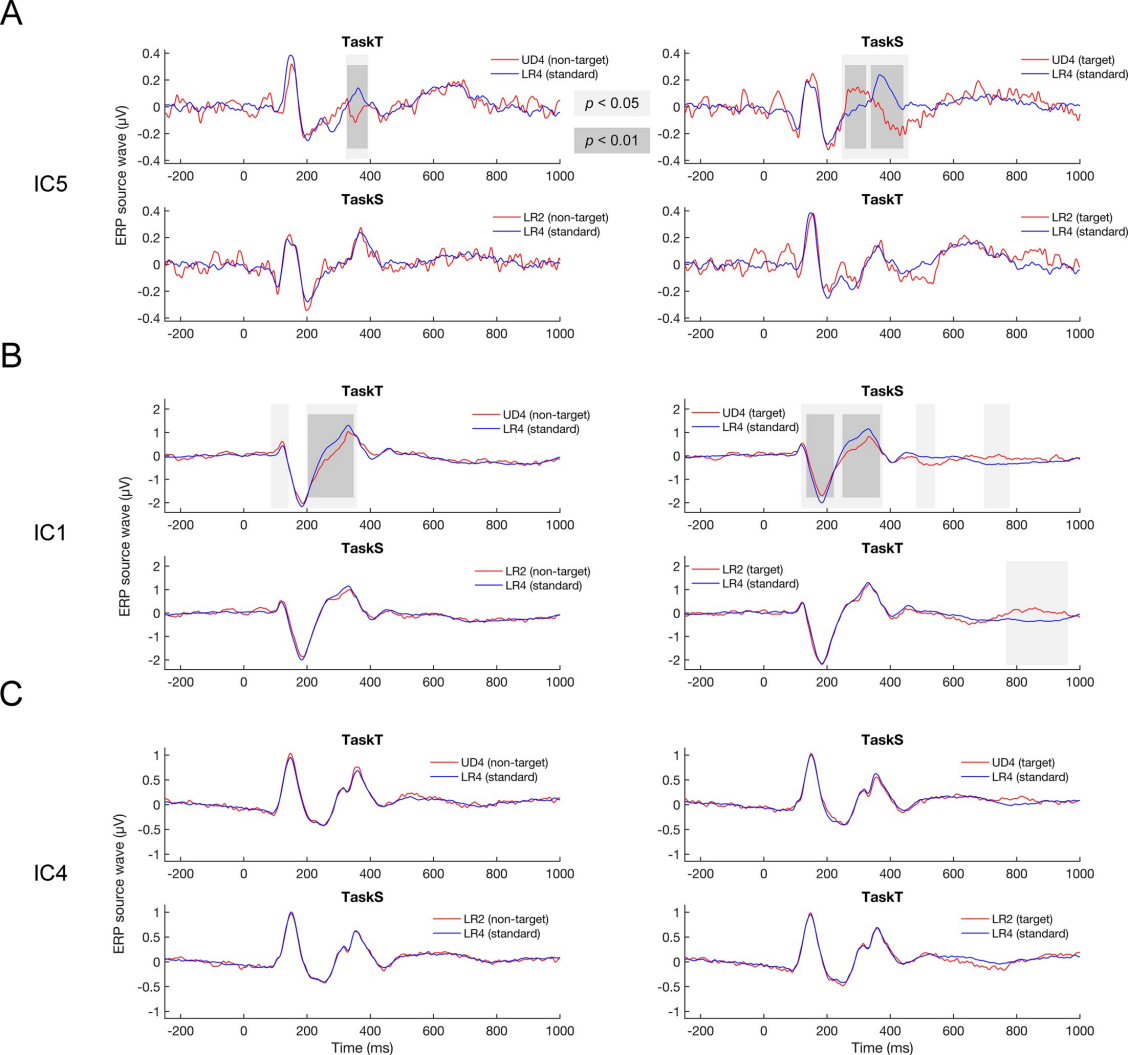
Responses arising from the visual cortex. (A) Significant differences occurred between IC5 to standard and non-target stimuli in TaskT, within the time range of 324–392 ms. Similar response differences were also observed in TaskS between IC5 to standard and target stimuli, within the time range of 334–456 ms. (B) Significant differences occurred between IC1 to standard and non-target stimuli in TaskT, within the time ranges of 88–140 ms and 200–356 ms. Similar response differences were also observed in TaskS between IC1 to standard and target stimuli, within the time ranges of 120–222 ms and 224–374 ms. In addition, significant differences occurred at a relatively late stage (later than 698 ms) between IC1 to standard and target stimuli in both TaskS and TaskT. (C) No difference was observed in either TaskT or TaskS between IC4 to standard and non-target stimuli or between IC4 to standard and target stimuli.

We observed significant differences between source waves arising from the visual cortex elicited by standard (LR4) and target deviant stimuli (UD4) in TaskS. The time range when the differences occurred was 248–332 ms (target deviant > standard) and 334–456 ms (target deviant < standard) for IC5 (Fig 5A), and 120–222 ms (target deviant > standard), 224–374 ms (target deviant < standard), 484–540 ms (target deviant < standard), and 698–776 ms (target deviant > standard) for IC1 (Fig 5B). No significant difference was found for IC4 in TaskS (Fig 5C). This suggests that the abstract structural pattern of Chinese characters could be extracted rapidly in the visual cortex when sufficient attentional resources were consciously devoted to the character structure. Also observed were significant differences between source waves arising from the visual cortex elicited by standard (LR4) and non-target deviant stimuli (UD4) in TaskT. The time range when the differences occurred was 324–392 ms (non-target deviant < standard) for IC5 (Fig 5A), and 88–140 ms (non-target deviant > standard) and 200–356 ms (non-target deviant < standard) for IC1 (Fig 5B). No significant difference was observed for IC4 in TaskT either (Fig 5C). This suggests that the visual cortex could rapidly detect violations of implicit abstract structural patterns even in the lack of conscious attention on structural features. The fact that there were significant differences for IC5 but not for IC4 suggests that this implicit extraction of abstract structural pattern of Chinese characters tended to be lateralized to the left primary/secondary visual cortices. The statistics of the mean amplitude of IC1 and IC5 within the time periods of interest were given in Table 4 and Table 5, respectively.

**Table 4:** Effects of IC1 reflecting extraction of abstract structural or tonal pattern.

Session	Time period (ms)	Character	Mean amplitude (μV)		paired-t
			mean	SD	(df = 15)
TaskS	120–222	LR4	–1.0444	0.6230	5.2597 ****
		UD4	–0.8270	0.5477	
	224–374	LR4	0.5884	0.4219	4.7262 ***
		UD4	0.2718	0.3412	
	484–540	LR4	–0.0440	0.4078	2.8416 *
		UD4	–0.3331	0.4594	
	698–776	LR4	–0.3569	0.3875	3.0693 **
		UD4	–0.0407	0.3320	
TaskT	88–140	LR4	0.1330	0.5515	3.4125 **
		UD4	0.2764	0.5796	
	200–356	LR4	0.2796	0.5139	6.1923 ****
		UD4	–0.0319	0.5815	
TaskT	768–960	LR4	–0.3161	0.4231	4.2771 ***
		LR2	0.0771	0.3011	

**** *p* < 0.0001

*** *p* < 0.001

** *p* < 0.01

* *p* <0.05.

**Table 5:** Effects of IC5 reflecting extraction of abstract structural pattern.

Session	Time period (ms)	Character	Mean amplitude (μV)		paired-t
			mean	SD	(df = 15)
TaskS	248–332	LR4	–0.0202	0.3313	5.4269 ****
		UD4	0.0930	0.3548	
	334–456	LR4	0.1043	0.1947	5.3730 ****
		UD4	–0.0881	0.2851	
TaskT	324–392	LR4	0.0719	0.3112	4.5840 ***
		UD4	–0.0333	0.3138	

**** *p* < 0.0001

*** *p* < 0.001

### Extraction of abstract tonal pattern: Source-level analyses

In contrast, there was no significant difference between source waves arising from the visual cortex elicited by standard (LR4) and target deviant stimuli (LR2) in TaskT, except the significant differences between IC1 elicited by standard (LR4) and target deviant stimuli (LR2) in the time range of 768–960 ms (target deviant > standard), which was quite late. There was no significant difference between source waves arising from the visual cortex elicited by standard (LR4) and non-target deviant stimuli (LR2) in TaskS (Fig 5B). These findings indicate that the rapid detection of violations of the abstract tonal pattern in the visual cortex was poor or even absent, whether the abstract tonal patterns were explicit or implicit to subjects. The statistics of the mean amplitude of IC1 within the time periods of interest were given in Table 4.

### Response of late higher-level processes: Source-level analyses

**Fig 6:**
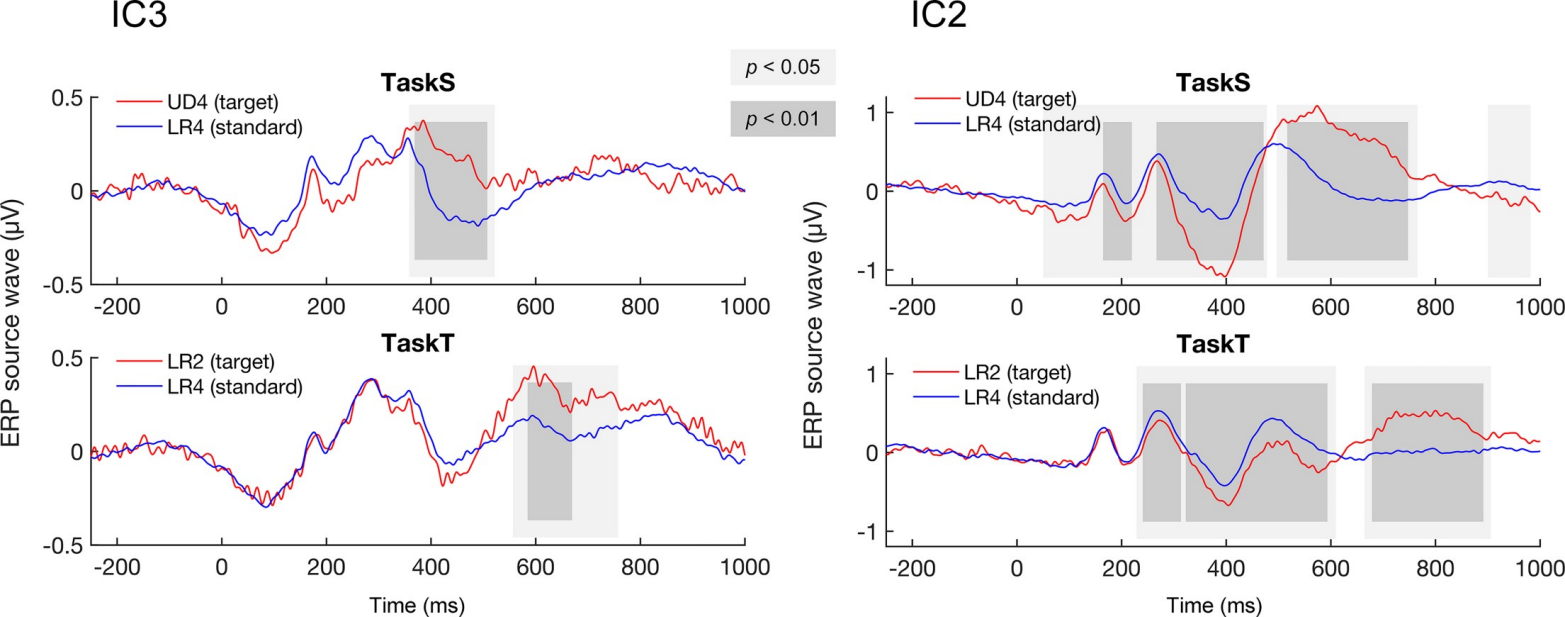
Responses representing higher-level processes. Significant differences occurred between IC3 to standard and target stimuli in both TaskS and TaskT. In addition, significant differences occurred between IC2 to standard and target stimuli in both TaskS and TaskT. Consistent with behavioral reaction time and IC1 results, response differences started earlier in TaskS than in TaskT for both IC3 and IC2.

When attentional resources were consciously and actively devoted to detect the target stimuli, there were significant differences between source waves representing higher-level processes (IC3 and IC2) elicited by standard and target deviant stimuli in both TaskS and TaskT. For IC3, whose sources were in the frontal lobe, the time range when the differences occurred was 360–520 ms (target deviant > standard) in TaskS and 558–756 ms (target deviant > standard) in TaskT (Fig 6). For IC2, which represented P300, the time range when the differences occurred was 52–476 ms (target deviant < standard), 498–764 ms (target deviant > standard), and 902–980 ms (target deviant < standard) in TaskS, and 230–608 ms (target deviant < standard) and 666–904 ms (target deviant > standard) in TaskT (Fig 6). This suggests involvement of attention-required higher-level processes in the extraction of explicit abstract patterns of both structural and tonal features of Chinese characters. Note that the response differences for the detection of target stimuli started much earlier in TaskS than in TaskT for both IC3 and IC2, consistent with behavioral performances. The statistics of the mean amplitude of IC2 and IC3 within the time periods of interest were given in Table 6 and Table 7, respectively.

**Table 6:** Effects of IC2 reflecting conscious detection of abstract structural or tonal pattern.

Session	Time period (ms)	Character	Mean amplitude (μV)		paired-t
			mean	SD	(df = 15)
TaskS	52–476	LR4	0.0046	0.1470	7.9903 ****
		UD4	–0.3170	0.2006	
	498–764	LR4	0.0868	0.1650	5.5241 ****
		UD4	0.7097	0.4397	
	902–980	LR4	0.0920	0.1866	2.5562 *
		UD4	–0.1073	0.2792	
TaskT	230–608	LR4	0.1135	0.2039	6.0362 ****
		LR2	–0.1003	0.2145	
	666–904	LR4	0.0075	0.1743	6.2637 ****
		LR2	0.3850	0.3239	

**** *p* < 0.0001

* *p* <0.05

**Table 7:** Effects of IC3 reflecting conscious detection of abstract structural or tonal pattern.

Session	Time period (ms)	Character	Mean amplitude (μV)		paired-t
			mean	SD	(df = 15)
TaskS	360–520	LR4	–0.0719	0.2183	4.9935 ***
		UD4	0.1938	0.1909	
TaskT	558–756	LR4	0.1197	0.1882	5.2062 ***
		LR2	0.3186	0.2635	

*** *p* < 0.001

Interestingly, we also observed significant differences between IC1 elicited by standard and target deviant stimuli at late stages of both TaskS and TaskT. The time range when the differences occurred was 484–540 ms (target deviant < standard), and 698–776 ms (target deviant > standard) in TaskS, and 768–960 ms (target deviant > standard) in TaskT (Fig 5). This suggests that late, attention-required processes in the visual cortex different from that underlying the extraction of implicit abstract patterns were engaged in the extraction of explicit abstract patterns, structural or tonal. When conscious attention was sufficiently devoted, the visual cortex was able to respond to violations of both abstract structural and tonal patterns in a relatively shorter time. Note that the late IC1 differences started much earlier in TaskS than in TaskT, consistent with behavioral performances and the results of IC2 and IC3. This consistency indicates that the late-response differences in the visual cortex that were specific to explicit abstract patterns might be relevant to top-down modulations of higher-level processes.

## Discussion

In this study, we investigated the possible extraction of abstract structural patterns of Chinese characters in the visual cortex, by examining whether the ERPs, especially the source waves arising from the visual cortex, changed in case of violations of explicit and implicit abstract structural patterns. Early response differences were observed between 88 and 456 ms after stimulus onset at different levels of the visual cortex, probably the left primary/secondary visual cortices and the bilateral associative visual cortices, whether the abstract structural pattern was explicit or implicit to subjects. On the other hand, we did not observe early response difference in the visual cortex for abstract tonal patterns. Moreover, response differences later than 698 ms after stimulus onset were also observed in the bilateral associative visual cortices for both explicit abstract structural and tonal patterns. These findings suggest that the visual cortex could rapidly extract abstract structural patterns of Chinese characters, whether or not conscious attention was focused on the target feature. Besides, the extraction of abstract tonal patterns was still feasible in the visual cortex at a relatively late stage if attentional resources were sufficiently devoted.

The differences between ERPs elicited by explicit and implicit changes in the abstract structural pattern were observed both at the sensor level and at the source level. In the sensor space, we selected occipital electrodes mainly because the responses of the visual cortex were of our primary interest. We observed ERP differences, most significantly in the time range of the P200 component, for both explicit and implicit violations of the abstract structural pattern, corresponding to the vMMN. However, there were still several other time ranges in which the ERP differences were also significant. Owing to the volume conduction of neurophysiological signals, it was not easy to convincingly attribute the ERPs recorded at these occipital electrodes to the brain region under them. That is to say, although we are confident to assume that the occipital ERPs mainly represents the neural activity of the visual cortex, especially at the latencies when typical visual ERP waves occurs, chances are that the scalp responses are dominated by cortices other than the visual cortex within some time periods. When ERPs in response to standard and deviant stimuli differed significantly, we relied on the topography of mean amplitude to examine whether the effects reflected certain processes that actually took place in the visual cortex or simply were products of volume conduction of processes that took place elsewhere. For example, the ERPs at occipital electrodes in response to LR4 and UD4 in TaskS differed around 638–756 ms, but it was almost unlikely that this difference effect reflected neural activities in the visual cortex, as the topographies of mean amplitude within this time range showed concentrated power around Pz, suggesting volume conduction of the P300 component. Therefore, it is necessary to conduct subsequent source-level analyses to clarify what brain regions the ERP difference effects could be attributed to and when these effects actually began and ended at their respective neural sources.

Using ICA-based space filters, we extracted three source waves arising from the visual cortex. Although the source reconstruction results based on dipole fitting and eLORETA were not exactly the same, they both supported that IC4 and IC5 arose from relatively lower levels of the visual cortex (probably primary/secondary visual cortices), whereas IC1 arose from higher levels of the visual system (probably bilateral associative visual cortices). We found evidence supporting that abstract structural patterns could be rapidly and implicitly extracted in the visual cortex, as a part of the orthographic processing of Chinese characters. We observed significant differences between IC1 in response to the standard (LR4) and the target deviant stimuli (UD4) in TaskS (before 374 ms). Moreover, significant differences between IC1 in response to the standard (LR4) and the non-target deviant stimuli (UD4) were also observed in TaskT (before 356 ms). Similar response differences were also observed for IC5 (before 456 ms), but not for IC4. These findings suggest that both explicit and implicit abstract structural patterns of Chinese characters can be extracted rapidly in the visual cortex, and that this rapid extraction is lateralized to the left hemisphere.

The sensory cortex is considered to have primitive or sensory intelligence that extracts abstract stimulus patterns from ongoing sequence [41]. The auditory cortex has been documented to have the primitive intelligence to extract the abstract patterns of Chinese lexical tones with various consonants and vowels [42]. In addition, the primitive intelligence to extract phonological features of Chinese characters is not limited to the auditory modality but also extends to the visual modality [31,32]. In this study, we add to the current literature that the visual cortex indeed has the primitive intelligence to extract abstract orthographic patterns, or to be more specific, abstract structural patterns. According to the predictive coding theory, different levels of the visual cortex are constantly predicting the responses generated at its lower level, and in case of any prediction error, an error signal is generated in order to correct the estimation of the input signal [43]. From this perspective, our findings suggest that the abstract structural pattern of upcoming Chinese characters is constantly predicted by associative visual cortices; when a violation occurs, the associative visual cortices will make a prediction error and concurrently the response differs from its normal form. A vital prediction of the predictive coding theory is the neuro-modulation of cells reporting prediction errors [44]. Consistently, we observed that the response differences to the standard (LR4) and the non-target deviant stimuli (UD4) in TaskT for IC1 occurred earlier than for IC5, whereas IC1 arose from levels higher than IC5 in the visual cortex. The later occurrence of response differences between standard and deviant stimuli at a lower level of the visual cortex seems to suggest that the abstract structural pattern of Chinese characters is not initially extracted at the primary level of the visual cortex, but that a top-down modulation from higher to lower levels of the visual cortex drives the response differences in IC5. Interestingly, only the left but not the right visual cortex is sensitive to violations of abstract orthographic patterns at the primary/secondary level, in line with previous literature that the left hemisphere is critical for orthographic processing [22], especially for recognition of Chinese characters [23]. These findings are consistent with the interactive model of visual word recognition where orthographic processing is considered to modulate low-level processing of visual inputs [45]. The corticofugal modulation seems to take place fast, possibly enabling the visual cortex to make quicker and better predictions about the current abstract pattern of character structure and thus adapting to the high-speed presentation of characters in real-life reading.

This primitive intelligence might be relevant to the processing underlying the emergence of position-specific and position-general radical representations. Previous literature documents that early radical representations are position-specific, but position-general radical representations also appears later [16], implying an abstracting process that extracts position information from initial radical representations. The current study provides supportive evidence that the abstract position information is indeed represented during orthographic processing and that the visual cortex is able to rapidly and implicitly detect this abstract pattern of the position information. Remarkably, the implicit extraction of abstract structural pattern documented in the current study seems to begin around the similar latencies of the emergence of position-specific radical representations (100–160 ms) and reaches the most significance around the similar latencies of the emergence of position-general ones (180–280 ms) [16]. Similarly in a study by Wei et al. [14], the authors reported vMMN effects within the time range of 120–160 ms for both real characters and pseudo-characters with legally-positioned radicals, but vMMN effects within the time range of 170–210 ms only for real characters. It is conceivable that position-specific radical representations should correctly emerge for both real characters and pseudo-characters, since the radicals forming the characters are all in their legal position. Therefore, their findings suggest that position-specific radical representations are indeed automatically extracted at an initial stage of Chinese character processing and that further lexical processing works for differentiation between real and pseudo-characters. Consistently, the most significant part of the vMMN effects reported in the current study seems to be partially overlapping, or even slightly later than, with the time range when the automatic extraction of position-specific radical representations (120–160 ms). The consistency of effect latency across multiple studies leads us to speculate that the extraction of abstract structural pattern documented in this study might share partially common mechanisms with the extraction of position-general radical representations, possibly via an abstracting process in the visual cortex that isolates abstract position information and representations of radical *per se*. Note that one advantage of the current study over previous ones is that we have provided evidence documenting where in the brain the ERP effects originated and pinpointed the neural sources underlying the ERP effects to the visual cortex. Despite the relatively poor spatial resolution of the ERP technique, the current study uses multiple source localization methods and obtains resembling results on the generating sites of the ERP difference effects, providing converging evidence in favor of the vital role of the visual cortex in the extraction of abstract structural patterns of Chinese characters during orthographic processing.

We did not observe evidence supporting the detection of violations of implicit abstract patterns of lexical tone in the visual system at low levels. This seems inconsistent with previous studies documenting a rapid, automatic detection of violations of abstract patterns of Chinese character phonology [31,32]. The inconsistency may be attributed to the different difficulties of tone identification tasks. In their study, they used Chinese characters with identical consonants and vowels that only varied in the lexical tone. But here in our study, we used Chinese characters with a variety of consonants and vowels as stimuli. Since it is more difficult for subjects to extract the lexical tone when consonants and vowels are more variable, the high difficulty of our tone identification task may impede the extraction of abstract tonal patterns. Another reasonable explanation is that the extraction of abstract phonological patterns in the visual cortex could reach only syllabic level but not deeper phonemic level. In other words, abstract syllabic patterns of Chinese characters can be rapidly and automatically extracted as documented by the studies by Wang et al. [31,32], but abstract phonemic patterns of Chinese characters may not be extracted in the visual cortex. According to the interactive model of visual word recognition, higher-order linguistic representations such as phonological representations can modulate early orthographic processing, which further modulates earlier visual processing [45]. It is conceivable that the effects of ERP differences (i.e. vMMN), if induced by top-down modulations, would be greater for the modulation of a product (syllable pronunciation) of the coarser part of phonological processing and weaker for the modulation of a product (phoneme) of the finer part of phonological processing. This may partially explain why we failed to observe any response difference in the visual cortex when violations of abstract tonal patterns occurred.

There are two limitations of this study that may affect the generalization of our findings. First, our use of an active oddball paradigm, together with the relatively long presentation time of the stimulus characters, renders it difficult for us to examine the role of attention in the extraction of the abstract structural pattern of Chinese characters. Although the dual-deviant oddball paradigm ensures that the ERP differences we found are not task-specific or target-specific, it is impossible for us to examine whether the rapid and implicit extraction of abstract structural patterns of Chinese characters was automatic and pre-attentive. After all, subjects paid conscious attention to all the characters in the character stream but focused on different features in different sessions. However, as an exploratory investigation, this study provides solid evidence supporting the implicit extraction of abstract structural patterns of Chinese characters. Whether this extraction is automatic and pre-attentive would be investigated in future studies using a passive oddball paradigm, e.g. a common passive paradigm used in vMMN studies where characters are presented peripherally and subjects pay attention to an irrelevant visual task at the center [24,32].

Second, it is not clear whether our findings may be generalized to structures of Chinese characters other than left-right structure or up-down structure. However, even if the results of this study failed to be generalized to other types of Chinese character structure and Chinese speakers could only differentiate characters with left-right structure from characters with up-down structure at the level of visual cortex, it would still be appropriate for us to conclude that the extraction of the abstract structural pattern of Chinese characters has already started in the visual cortex. The core question here is what caused the ERP differences we observed in this study. They cannot be induced due to the differences in familiarity of the characters, as we controlled the occurrence frequency of the stimulus characters to ensure they were matched across stimulus types. We cannot rule out the possibility that they are induced by the differences in familiarity of the abstract structural patterns. This could be examined in future studies where the structure familiarity difference between standard and deviant stimuli is manipulated so that it could be examined whether the ERP differences change with structure familiarity correspondingly. However, since it is likely that the visual system of Chinese speakers learns to extract the abstract structural pattern of Chinese characters during their long-term linguistic experience, it is possible that the differences in occurrence frequency of various structures determine, at least partially, the learning at the visual cortex and consequently the exact extraction strategy heavily depends on the familiarity.

In conclusion, this study documents that the visual cortex is able to extract abstract patterns of spatial arrangement of radicals (i.e. structure) of Chinese characters rapidly, no matter the abstract pattern is explicit or implicit to readers. The left primary/secondary visual cortices and the bilateral associative visual cortices are probably involved in this left-lateralized extraction. Nevertheless, we have not found evidence in favor of a similar rapid extraction in the visual cortex of abstract patterns of the lexical tone of Chinese characters. These findings suggest that the primitive intelligence of the visual cortex may be restricted to extract abstract patterns of limited features of Chinese characters.

## Abbreviations

EEG: electroencephalography
ERP: event-related potential
FDR: false discovery rate
ICA: independent component analysis
RV: residue variance
vMMN: visual mismatch negativity
